# *Artemisia princeps* Inhibits Biofilm Formation and Virulence-Factor Expression of Antibiotic-Resistant Bacteria

**DOI:** 10.1155/2015/239519

**Published:** 2015-07-12

**Authors:** Na-Young Choi, Sun-Young Kang, Kang-Ju Kim

**Affiliations:** ^1^College of Education, Wonkwang University, Iksan 570-749, Republic of Korea; ^2^Department of Oral Biochemistry, School of Dentistry, Wonkwang University, Iksan 570-749, Republic of Korea; ^3^Wonkwang Research Institute for Food Industry, Iksan 570-749, Republic of Korea; ^4^Department of Oral Microbiology, School of Dentistry, Wonkwang University, Iksan 570-749, Republic of Korea

## Abstract

In this study, we used ethanol extract of *A. princeps* and investigated its antibacterial effects against MRSA. Ethanol extract of *A. princeps* significantly inhibited MRSA growth and organic acid production during glucose metabolism at concentrations greater than 1 mg/mL (*P* < 0.05). MRSA biofilm formation was observed using scanning electron microscopy (SEM) and safranin staining. *A. princeps* extract was found to inhibit MRSA biofilm formation at concentrations higher than 2 mg/mL significantly (*P* < 0.05). Bactericidal effects of the *A. princeps* were observed using confocal laser microscopy, which showed that *A. princeps* was bactericidal in a dose-dependent manner. Using real-time PCR, expression of *mecA*, an antibiotic-resistance gene of MRSA, was observed, along with that of *sea, agrA*, and *sarA*. *A. princeps* significantly inhibited *mecA, sea, agrA*, and *sarA*, mRNA expression at the concentrations greater than 1 mg/mL (*P* < 0.05). The phytochemical analysis of *A. princeps* showed a relatively high content of organic acids and glycosides. The results of this study suggest that the ethanol extract of *A. princeps* may inhibit proliferation, acid production, biofilm formation, and virulence gene expressions of MRSA, which may be related to organic acids and glycosides, the major components in the extract.

## 1. Introduction

The appearance of antibiotic-resistant bacteria has led to difficulties in treating infectious diseases. Therefore, new antibiotics have been developed, but bacteria rapidly evolve resistance to the new treatments, neutralizing their efficacy. Methicillin-resistant* Staphylococcus aureus* (MRSA) is one of such resistant strains.* S. aureus* is a bacterium commonly observed in nature as well as in normal skin, the nasal cavity, and the oral cavity. The bacterium causes diseases in many organs, including the skin when an individual is immunocompromised; this can result in serious diseases such as endocarditis and septicemia [1].

When antibiotics such as penicillin were developed, treatments for* S. aureus* infection were effective because of the high sensitivity of the bacterium to antibiotics; however, the frequency of resistant bacteria has increased with the utilization of penicillin [2, 3]. The bacteria show resistance by producing *β*-lactamase and inactivating antibiotics such as penicillin. Subsequently, methicillin, which is not inactivated by *β*-lactamase, was developed and utilized for treating penicillin-resistant bacteria. However, since MRSA emerged in the early 1960s, its frequency has gradually increased [4]. MRSA is resistant to not only *β*-lactamase antibiotics but also many other antibiotics, making effective treatment difficult [5, 6]. Mechanisms of MRSA antibiotic resistance are not clearly understood. However, it is known that MRSA possesses* mecA* genes and produces PBP 2′, a derivative of penicillin-binding protein (PBP), which reduces the affinity of PBP to *β*-lactamase antibiotics such as methicillin. In addition, resistance is reported to occur through the acquisition of resistant plasmid (R plasmid) with *β*-lactamase-expressing genes [2]. MRSA can form biofilms in implants that are injected into patients, in medical supplies or in medical devices [7, 8]. Once biofilms are formed, they cannot be easily neutralized by antibiotics, paving the way to serious pathogenic infection [9]. MRSA can produce organic acids via carbohydrate metabolism pathways. It has been reported that organic acids produced by MRSA decrease pH and can facilitate biofilm formation [10, 11]. MRSA possesses several virulence factors:* sea* encodes staphylococcal enterotoxin A (SEA). SEA, one of major virulence factors of* S. aureus* [12], is known to induce staphylococcal gastroenteritis, secrete T-cell-derived cytokines, and stimulate T-cell activation owing to the presence of immunomodulatory properties of superantigens. In* S. aureus*, production of virulence factors is regulated by global regulators such as* agr* and* sarA* [13].

MRSA has emerged to be one of the most important pathogenic bacteria because of its characteristics such as multidrug resistance and biofilm formation. Therefore, new drugs capable of treating an MRSA infection must be developed. Historically, many scientists have identified antibacterial substances in natural products, which is currently an active field of study [14, 15].

*Artemisia princeps* (*A. princeps*), a perennial herb belonging to the Asteraceae family, is distributed throughout East Asia and is widely used to maintain hemostasis and in treating pain, hypermenorrhea, amenorrhea, uterine hemorrhage, hemorrhoids, inflammation, and menopausal diseases [16].

However, few studies have examined the effects of* A. princeps* on MRSA. In studies conducted to discover natural products with antibacterial effects against MRSA, it was found that ethanol extract of* A. princeps* showed antibacterial effects against MRSA. In this study, we investigated the inhibitory effect of* A. princeps* on proliferation, acid production, biofilm formation, and virulence gene expression of MRSA. Phytochemical analyses were also performed to investigate detailed chemical constituents of ethanol extract of* A. princeps*.

## 2. Materials and Methods

### 2.1. Material

Brain heart infusion (BHI) broth was purchased from Difco Laboratories (Detroit, MI, USA). Glucose and dimethyl sulfoxide (DMSO) were obtained from Sigma Co. (St. Louis, MO, USA). MRSA ATCC 33591 was purchased from the American Type Culture Collection (ATCC, Manassas, VA, USA).

### 2.2. Plant Material and Extraction

The leaves of* A. princeps* were obtained from the oriental drug store Dae Hak Yak Kuk (Iksan, South Korea). The identity of the specimen was confirmed by Young-Hoi Kim at the College of Environmental and Bioresource sciences, Chonbuk National University (Jeonju, South Korea). A voucher specimen (number 8-10-13) has been deposited at the Herbarium of the Department of Oral Biochemistry in Wonkwang University. Dried leaves (250 g) of* A. princeps* were soaked in 3,500 mL of 70% ethanol for 72 h at room temperature. The extracted solution was filtered and evaporated under reduced pressure to yield an ethanol extract of 5.7 g (2.28%). After the extract was thoroughly dried to facilitate complete removal of the solvent, the dry extract was dissolved in DMSO to give the desired stock solution. The final concentration of DMSO applied to culture systems was adjusted to 0.1% (v/v), which did not interfere with the testing system. Control groups were treated with media containing 0.1% DMSO.

### 2.3. Bacterial Growth and Acid Production

Bacterial growth was determined using a modification of a previously described method [3, 8]. The growth of MRSA was examined at 37°C in 0.95 mL of BHI broth containing various concentrations of the ethanol extract of* A. princeps*. These tubes were inoculated with 0.05 mL of an overnight culture grown in BHI broth [final: 5 × 10^5^ colony-forming units (CFU)/mL] and incubated at 37°C. After 24 h of incubation, the optical density (OD) of cells was measured spectrophotometrically at 550 nm, and the pH of the cultures was determined using a pH meter (Corning Inc., Corning, NY, USA). Three replicates were measured for each concentration of the test extract. NaF (0.1%) was used as a positive control.

### 2.4. Biofilm Assay

The biofilm assay was based on a method described previously [6, 11].* A. princeps* extract was added to BHI broth containing 1% glucose in 35 mm polystyrene dishes, or 24-well plates (Nunc, Copenhagen, Denmark). The cultures were then inoculated with a seed culture of MRSA (final: 5 × 10^5^ CFU/mL). After cultivating for 48 h at 37°C, the supernatant was removed completely, and the dishes, wells, or wells containing resin teeth were rinsed with distilled water. The amount of biofilm formed in the wells was measured by staining with 0.1% safranin. The bound safranin was released from the stained cells with 30% acetic acid, and the absorbance of the solution was measured at 530 nm. The biofilm formed on the surface of the resin teeth was also stained with 0.1% safranin and photographed.

### 2.5. Scanning Electron Microscopy (SEM)

The biofilm on 35 mm polystyrene dishes was also determined by SEM using a modification of a previously described method [4]. The biofilm formed on the dishes was rinsed with distilled water and fixed with 2.5% glutaraldehyde in 0.1 M sodium cacodylate buffer (pH 7.2) at 4°C for 24 h. After gradual dehydration with ethyl alcohol 60, 70, 80, 90, 95, and 100%, the sample was freeze-dried. The specimens were then sputter-coated with gold (108A sputter coater, Cressington Scientific Instruments Inc., Watford, UK). For observation, a JSM-6360 SEM (JEOL, Tokyo, Japan) was used.

### 2.6. Confocal Laser Scanning Microscopy

Bactericidal effect of* A. princeps* extract was determined by confocal laser scanning microscopy. The cultured MRSA in BHI was diluted using BHI media to approximately 1 × 10^7^ CFU/mL. The bacteria (1 × 10^7^ CFU/mL) were treated with high concentrations (8–64 mg/mL) of* A. princeps* extract at 37°C under aerobic conditions. After 30 min of incubation, the bacteria were washed with PBS and stained with LIVE/DEAD BacLight Bacterial Viability Kit (Molecular Probes, Eugene, OR, USA), prepared according to the manufacturer's instructions, for 15 min. Stained bacteria were observed confocal laser scanning microscopy (LSM 510, Zeiss, Germany). This method is based on two nucleic acid stains: green fluorescent SYTO 9 stain and red fluorescent propidium iodide stain which differ in their ability to penetrate healthy bacterial cells. SYTO 9 stain labels live bacteria, in contrast propidium iodide penetrates only bacteria with damaged membranes.

### 2.7. Real-Time Polymerase Chain Reaction (PCR) Analysis

To determine the effect of* A. princeps* extract on gene expression, a real-time PCR assay was performed. The subminimal inhibitory concentration (1–4 mg/mL) of* A. princeps* extract was used to treat and culture MRSA for 24 h. Total RNA was isolated from* S. mutans* by using Trizol reagent (Gibco-BRL) according to the manufacturer's instructions. Then, cDNA was synthesized using a reverse transcriptase reaction (Superscript; Gibco-BRL). The DNA amplifications were carried out using an ABI-Prism 7,000 Sequence Detection System with Absolute QPCR SYBR Green Mixes (Applied Bio systems Inc., Foster City, CA, USA). The primer pairs that were used in this study were described by previous report [17–20] and are listed in Table 1. 16S rRNA was used as an internal control.

### 2.8. Phytochemical Screening

Phytochemical tests of the extract were performed as previously described [21, 22]. Mayer's reagent was used for alkaloids, ferric chloride reagent for phenolics, Molish test for glycosides, Biuret reagent for peptides, Mg-HCl reagent for flavonoids, Liebermann-Burchard reagent for steroids, and silver nitrate reagent for organic acids.

### 2.9. Statistical Analysis

All experiments were performed in triplicate. Data were analyzed using the Statistical Package for Social Sciences (SPSS, Chicago, IL, USA). The data are expressed as the mean ± standard deviation values. The differences between the means of the experimental and control groups were evaluated by Student's *t*-test. Values of *P* < 0.05 were considered statistically significant.

## 3. Results

**Figure 1:**
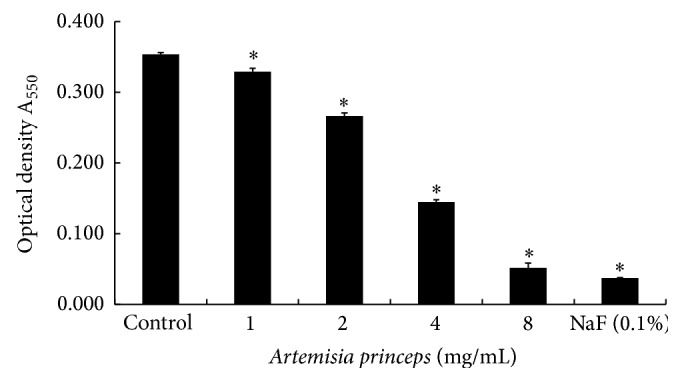
Effect of ethanol extract of* A. princeps* on the growth of MRSA. MRSA was inoculated into BHI broth with various concentrations of* A. princeps* and incubated for 24 h at 37°C. The optical density (A_550_) was read using a spectrophotometer. Data are mean ± standard deviation. ^*^ *P* < 0.05 compared to the control group.

In the present study, after performing ethanol extraction of* A. princeps*, the antibacterial effects against MRSA were tested. The results are shown in Figure 1. After treating MRSA with 1, 2, 4, and 8 mg/mL of ethanol extract of* A. princeps* showed a dose-dependent manner the antibacterial effects of* A. princeps* ethanol extract against MRSA were observed. Compared to controls, the ethanol extract of* A. princeps* showed significant inhibition of MRSA growth at concentrations higher than 1 mg/mL. The positive control used in this study, 0.1% NaF, also showed antibacterial effects. The minimum inhibitory concentration (MIC) of ethanol extract of* A. princeps* against MRSA was confirmed to be 8 mg/mL. These results indicate that the ethanol extract of* A. princeps* has antibacterial effects against MRSA.

MRSA generates organic acids by metabolizing carbohydrates. To investigate whether the ethanol extract of* A. princeps* can suppress organic acid production in MRSA, the ethanol extract of* A. princeps* was added to MRSA culture medium and the change in pH was measured. The pH of the control culture medium was approximately 7.2 before the incubation, which decreased to approximately 5.87 after culturing (Table 2). However, this decrease in pH was suppressed in the group treated with the ethanol extract of* A. princeps* (1–8 mg/mL). The positive control, 0.1% NaF, also showed suppression in pH reduction. These results reveal that organic acid production in MRSA can be inhibited by the ethanol extract of* A. princeps*.

**Figure 2:**
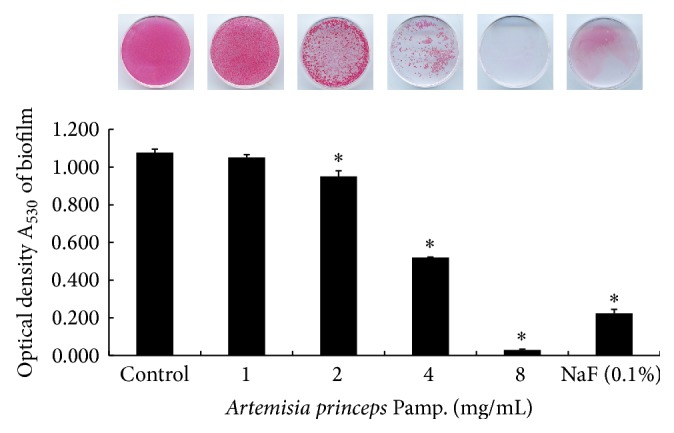
Effect of ethanol extract of* A. princeps* on biofilm formation by MRSA. MRSA was inoculated into BHI broth with various concentrations of* A. princeps* and incubated for 48 h at 37°C. The biofilms that formed on the dish surface were measured by staining with 0.1% safranin. The bound safranin was released from the stained cells with 30% acetic acid, and the absorbance of the solution was measured at 530 nm. Data are represented as mean ± standard deviation. ^*^ *P* < 0.05 compared to the control group.

**Figure 3:**
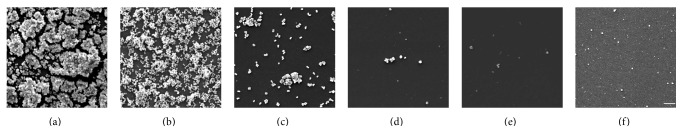
Scanning electron microscopy of MRSA biofilms grown in ethanol extract of* A. princeps*. (a) Control; (b) 1 mg/mL; (c) 2 mg/mL; (d) 4 mg/mL; (e) 8 mg/mL; (f) positive control (0.1% NaF); Bar = 10 *μ*m.

MRSA forms biofilms on implants, medical supplies, or on medical devices, which eventually increases the bacterium's antibiotic resistance. In this study, we examined whether an ethanol extract of* A. princeps* was able to inhibit the biofilm formation of MRSA. Using safranin staining, the ethanol extract of* A. princeps* at concentrations of 2–8 mg/mL was seen to inhibit MRSA biofilm formation (Figure 2). The positive control used in this study, 0.1% NaF, also inhibited biofilm formation. These results were confirmed by SEM images (Figure 3), which showed similar outcomes to the results of safranin staining. In the control that was not treated with the ethanol extract of* A. princeps*, MRSA adhered densely to the surface of the polystyrene 35 mm dish and formed biofilms, whereas biofilm formation decreased in proportion to the ethanol extract concentration when the extract was introduced. Biofilm formation also decreased in the positive control.

**Figure 4:**
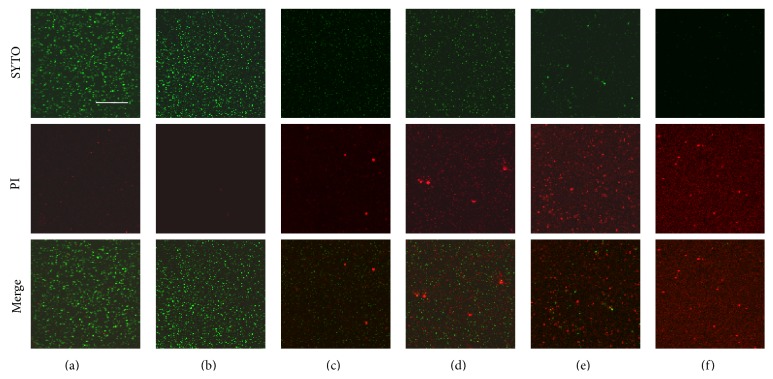
Bactericidal effect of ethanol extract of* A. princeps*. Cultured MRSA was treated with high concentration (8–64 mg/mL) of* A. princeps* extract and stained with LIVE/DEAD BacLight Bacterial Viability Kit. The stained bacteria were observed confocal laser scanning microscopy. Treatment with ethanol extract of* A. princeps* decreased green-labeled living bacteria (SYTO 9 stain) and increased red-labeled dead bacteria (PI stain) in a dose-dependent manner. (a) Control; (b) 8 mg/mL; (c) 16 mg/mL; (d) 32 mg/mL; (e) 64 mg/mL; (f) positive control (0.1% NaF); Bar = 50 *μ*m.

**Figure 5:**
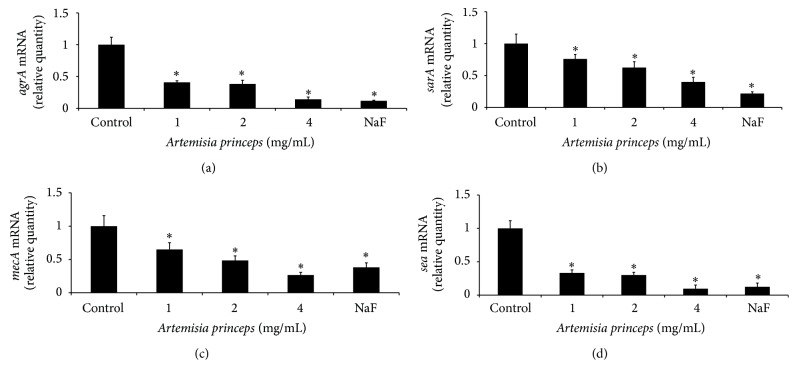
Real-time PCR analysis of expression of several virulence-factor genes. MRSA was cultured and treated with subminimal inhibitory concentration (1–4 mg/mL) of* A. princeps* extract, and real-time PCR analysis was then performed as described in Section 2. Expression of* mecA*,* sea*, and* agrA* was significantly inhibited at concentration higher than 1 mg/mL. Each value is expressed as a mean ± standard deviation. Significance was determined at ^*^ *P* < 0.05 when compared with the control.

Using a confocal laser microscopy, the bactericidal effects of ethanol extract of* A. princeps* were examined. The ethanol extract of* A. princeps* was observed to be bactericidal in a dose-dependent manner (8–64 mg/mL) (Figure 4). Expression of* mecA*, an antibiotic-resistant gene of MRSA, as well that of* sea, agrA*, and* sarA* and virulence-factor genes was estimated using real-time PCR in sub-MIC. The ethanol extract of* A. princeps* inhibited* mecA*,* sea*,* agrA,* and* sarA* mRNA expression at concentrations greater than 1 mg/mL (Figure 5).

Phytochemical analysis of* princeps* showed relatively high organic acid content, medium glycosides content, and weak phenolic content. Alkaloid, flavonoid, and peptides were detected only at very low levels (Table 3).

## 4. Discussion

MRSA is a typical antibiotic-resistant strain. Development of new antibiotics is necessary for treating such strains. Natural products can be utilized as raw materials in the development of new antibacterial substances.

*A. princeps* is used as a digestive medicine, in treating fever, as an anthelmintic, and as an antihemorrhagic agent in oriental medicines and is known to be effective for treating gynecological and gastrointestinal diseases. In addition, its constituents are known to have various physiological effects such as insecticidal, antibacterial, and antitumor effects [23, 24].

In this study, we investigated the antibacterial effects of an ethanol extract of* A. princeps* against MRSA. The ethanol extract of* A. princeps* at 1–8 mg/mL was found to inhibit MRSA growth. The evidence of antibacterial effects of the ethanol extract of* A. princeps* supports the use of* A. princeps* as a traditional medicine to treat patients with infectious diseases in Korea. According to previous studies on the aroma constituents of* A. princeps*, thujone, caryophyllene, and farnesol showed antibacterial effects against* Escherichia coli, Enterobacter aerogenes, Vibrio parahaemolyticus, Pseudomonas aeruginosa, Bacillus subtilis,* and* Staphylococcus aureus* [23].

MRSA is known to produce organic acids through carbohydrate metabolism pathways [10, 11]. Acetic acid is the main organic acid produced by MRSA, which lowers pH in the infected area; this lowered pH facilitates biofilm formation by microorganisms [10]. Our results showed that the ethanol extract of* A. princeps* inhibited the pH reduction induced by MRSA. This result indicates that the ethanol extract of* A. princeps* inhibits carbohydrate metabolism in MRSA.

MRSA adheres to and proliferates in damaged tissue, implanted medical and prosthetic devices, and is capable of forming biofilms [7, 8]. Biofilms constitute bacterial communities that form on the surface of living and nonliving substances. Biofilms are surrounded by a self-produced extracellular matrix that consists of polysaccharides and proteins. These biofilms are very difficult to remove and are a cause of intractable infection. Biofilms formed on the surface of implanted medical devices cannot be removed by antibiotic injection and can only be removed by surgery in most cases. After excising the peripheral tissues of biofilms, antibiotics need to be utilized for a long period of time [7, 8]. The most well-known method for analyzing biofilm formation is a tissue culture plate assay method [9]. In this study, biofilm formation was observed via safranin staining. The result showed that* A. princeps* inhibited biofilm formation of MRSA at concentrations of 1–8 mg/mL. Similar results were observed when MRSA biofilm formation was measured by SEM. A previous study reported that biofilm culture and planktonic culture showed different physiological characteristics, even when the same types of bacteria were cultured. Biofilm formation reportedly increases the immune response of bacteria and their resistance to antibacterial substances [17]. However, comparing biofilm culture data and planktonic culture data in the present study, there were no considerable differences in their resistance to ethanol extract of* A. princeps* between the two groups.

Bactericidal effects of ethanol extract of* A. princeps* were observed using confocal laser microscopy; the ethanol extract* A. princeps* showed bactericidal activity in a dose-dependent manner.

In MRSA, PBP 2′ produced by the* mecA* gene has low affinity to *β*-lactams antibiotics such as methicillin, and it is known to be antibiotic-resistant for cell-wall synthesis, even in the presence of *β*-lactamase antibiotics [2]. In this study,* mecA* expression, an antibiotic-resistant gene of MRSA, was examined using real-time PCR. The ethanol extract of* A. princeps* inhibited* mecA* expression at concentrations higher than 1 mg/mL.

In MRSA,* sea* encodes SEA, which is a major virulence factor of* S. aureus* [12]. SEA induces staphylococcal gastroenteritis, secretion of T-cell-derived cytokines and T-cell activation because SEA possesses immunomodulatory properties of superantigens. The ethanol extract of* A. princeps* was observed to inhibit* sea* expression at concentrations higher than 1 mg/mL.

Production of virulence factors in* S. aureus* is controlled by global regulators such as* agr* and* sarA* [13].* agrA* encodes accessory gene regulator A; when* agrA* expression is inhibited, production of virulence factors is also inhibited. In addition,* sarA* regulates the production of some matrix adhesion genes (e.g.,* fnbA*) and exotoxin genes (e.g.,* hla*), the virulence factors associated with adherence of* S. aureus* [25]. Our investigation of the expression of the* agrA* and* sarA* genes in this study showed that* A. princeps* inhibited* sea* expression at concentrations higher than 1 mg/mL.

According to a previous study,* Artemisia *spp. contain many components with strong antioxidant effects, such as caffeic acid, catechol, protocatechuic acid, vanillin, umbelliferone, and ferulic acid [26].

Moreover, cineol, thujone, caryophyllene, humulene, linalool, artemisia alcohol, camphor, farnesol, and borneol are present in* A. princeps*; tetracosanol, *β*-sitosterol, l-chebulachitol, and l-inositol are present in the organism's leaves, with caryophyllene and farnesol known to have antibacterial effects.

Based on our phytochemical analysis in the present study, the ethanol extract of* A. princeps* was confirmed to contain organic acids, glycosides, and phenolics. In particular, organic acids and glycosides showed an intense reaction and are thought to be the active components in the MRSA inhibition mechanisms. Therefore, further studies to examine the specific effects of the components present in* A. princeps* are necessary. Furthermore, additional studies should be conducted to identify antibacterial substances against MRSA.

In conclusion, we show that the ethanol extract of* A. princeps* may inhibit proliferation, acid production, biofilm formation, and virulence gene expressions of MRSA, which may be related to organic acids and glycosides, the major components in the extract of* A. princeps*.

## Figures and Tables

**Table 1:** Nucleotide sequences of primer used for real-time PCR in this study.

Gene	Gene description	Primer sequences (5′-3′)	
		Forward	Reverse
*16srRNA *	Normalizing internal standard	ACTGGGATAACTTCGGGAAA	CGTTGCCTTGGTAAGCC
*mecA *	Penicillin binding protein 2′	GTTAGATTGGGATCATAGCGTCATT	TGCCTAATCTCATATGTGTTCCTGTAT
*Sea *	Staphylococcal enterotoxin A	ATGGTGCTTATTATGGTTATC	CGTTTCCAAAGGTACTGTATT
*agrA *	Accessory gene regulator A	TGATAATCCTTATGAGGTGCTT	CACTGTGACTCGTAACGAAAA
*sarA *	Staphylococcal accessary regulator A	TGTTATCAATGGTCACTTATGCTG	TCTTTGTTTTCGCTGATGTATGTC

**Table 2:** Effect of ethanol extract of *A*. *princeps* on acid production by MRSA.

Conc. (mg/mL)	pH (before incubation)	pH (after incubation)
Control	7.20 ± 0.00	5.87 ± 0.00
1	7.20 ± 0.00	6.12 ± 0.04^*^
2	7.20 ± 0.00	6.65 ± 0.02^*^
4	7.20 ± 0.00	6.79 ± 0.00^*^
8	7.20 ± 0.00	7.10 ± 0.00^*^
0.1% NaF	7.20 ± 0.00	7.07 ± 0.05^*^

Date (pH) are represented as mean ± standard deviation.

^*^ *P* < 0.05 when compared with the control group after incubation.

**Table 3:** Phytochemical analysis of the ethanol extract of *Artemisia princeps*.

Plant constituents	Ethanol extract
Alkaloids	−
Phenolics	+
Flavonoids	−
Glycosides	++
Peptides	−
Steroids, terpenoids	−
Organic acids	+++

+++: strong; ++: moderate; +: poor; −: absent.
